# The Antioxidant, Cytotoxic and Antimicrobial Potential of Phenolic Acids-Enriched Extract of Elicited Hairy Roots of *Salvia bulleyana*

**DOI:** 10.3390/molecules27030992

**Published:** 2022-02-01

**Authors:** Marta Krzemińska, Aleksandra Owczarek, Weronika Gonciarz, Magdalena Chmiela, Monika A. Olszewska, Izabela Grzegorczyk-Karolak

**Affiliations:** 1 Department of Biology and Pharmaceutical Botany, Medical University of Lodz, Muszynskiego 1, 90-151 Lodz, Poland; marta.wojciechowska2@stud.umed.lodz.pl; 2 Department of Pharmacognosy, Medical University of Lodz, Muszynskiego 1, 90-151 Lodz, Poland; aleksandra.owczarek@umed.lodz.pl (A.O.); monika.olszewska@umed.lodz.pl (M.A.O.); 3 Department of Immunology and Infectious Biology, Faculty of Biology and Environmental Protection, University of Lodz, Banacha 12/16, 90-237 Lodz, Poland; weronika.gonciarz@biol.uni.lodz.pl (W.G.); magdalena.chmiela@biol.uni.lodz.pl (M.C.)

**Keywords:** anethole, cadmium chloride, elicitation, methyl jasmonate, phenolic acids, rosmarinic acid, yeast extract

## Abstract

Hairy root cultures are valuable sources of a range of phytochemicals. Among them, *Salvia bulleyana* root culture is a promising source of polyphenols, especially rosmarinic acid (RA), a phenolic acid depside with pleiotropic activity and a wide application in medicine and cosmetology. The aim of the study was to enhance the culture productivity by finding suitable elicitation protocol and to determine its biological potential in terms of antioxidant, anticancer and antimicrobial properties. The total content of phenols and the levels of particular constituents in root extracts were analyzed using HPLC-PDA. Among four elicitors tested (yeast extract; methyl jasmonate, MJA; trans-anethol; and cadmium chloride), MJA was found to be the most effective. The greatest boost in phenolic production (up to 124.4 mg/g dry weight) was observed after three-day treatment with MJA at 100 µM, with an almost 100% improvement compared to the controls (non-treated root culture). The hydromethanolic extract from the elicited culture exhibited strong antioxidant activity with IC_50_ values of 11.1 µg/mL, 6.5 µg/mL and 69.5 µg/mL for DPPH (2,2-diphenyl-1-picrylhydrazyl), ABTS (2,2-azinobis-(3-ethylbenzthiazoline-6-sulfonic acid)) and superoxide anion radical, respectively. Moreover, in concentrations of 0.5–5 mg/mL the extract inhibited the growth of LoVo, AGS and HeLa cell lines, but was safe for the L929 cells up to the concentration of 5 mg/mL. The extract also exhibited moderate antimicrobial activity. Thus, the results confirmed that elicitation can be a beneficial strategy for increase the phenolic acid biosynthesis in hairy roots of *S. bulleyana*, and that such a highly productive culture can show significant biological potential.

## 1. Introduction

Phenolic acids are natural plant metabolites known for their numerous biological activities. Reports published in the last 20 years suggest that they can facilitate treatment of civilization diseases, including those caused by oxidative damage to tissues and organs and age- and environmental-related inflammatory processes [1,2,3]. Some of the phenolic acids can also prevent and limit the growth of cancer, and can be used to fight microorganisms in the face of progressive antibiotic resistance [1,2,4]. One of the better-known compounds from this group is rosmarinic acid (RA), an ester of caffeic and 3,4-dihydroxyphenyllactic acids. It is an effective antioxidant that can scavenge free radicals, chelate pro-oxidant ions and protect cell membranes from damage by preventing lipid peroxidation [1]. It thus exerts neuroprotective, hepatoprotective and cardioprotective effects [2]. In addition, in vitro and in vivo studies have found RA to exhibit anti-inflammatory [3] and anticarcinogenic activities [4,5]. 

The compound has also demonstrated antimicrobial potential. It is known to damage the surface of fungal hyphae by physically disrupting the cell wall [6]. Moreover, it has been found to possess an antibacterial effect against numerous wild strains of Gram-positive and Gram-negative bacteria and a synergistic effect with some antibiotics against antibiotic-resistant strains [7]. RA has also been shown to possess antiviral activity by inhibiting the entry of viruses into host cells and reducing viral replication [8,9]. Additionally, recent research indicates that it exhibits binding affinity for SARS-CoV-2 viral protein targets [10].

RA is a metabolite commonly found in the *Lamiaceae* family, including numerous species of the genus sage. However, the quantity of the phenolic acid in natural or cultivated *Salvia* roots is relatively low and variable [11]. Therefore, to provide an alternative source for stable, year-round production of RA and other bioactive polyphenols, interest has grown in the use of plant cells and organ cultures. One exceptionally valuable source of RA is *S. bulleyana* hairy root culture [12]. This perennial grows, among others, in Yunnan Province, China, where it is used as an alternative to *danshen*: *Salvia miltiorrhiza* root [13]. Our previous biotechnological studies have resulted in obtaining highly productive, transformed *S. bulleyana* roots [12]. Apart from rosmarinic acid, this culture turned out to be an important source of other, less common secondary metabolites such as salvianolic acid K (SAK), E (SAE) or two isomers of salvianolic acid F (SAF I, SAF II). Moreover, the culture was rapidly growing and stable; therefore, it is a suitable starting material for further enhancement [12].

The use of plant culture systems offers many ways to support and improve the production of bioactive compounds by optimizing medium composition and culture conditions, precursor feeding or elicitation [14]. Elicitors are stress factors that play an important role in adaptation to stressful conditions by triggering various defense responses in plants, including the production and accumulation of secondary metabolites. This has been earlier reported for different phytochemicals known for their pharmacological activity, such as alkaloids, cardenolides, terpenoids and phenols [15,16,17]. 

Some studies have examined the use of elicitors in in vitro sage cultures; however, most have focused on *S. miltiorrhiza* [18,19,20,21], with only a few examining other species, such as *S. przewalskii*, *S. virgata*, *S. officinalis* and *S. castanea* [22,23,24]. These studies are usually limited to one or two elicitors, but their results seem promising. Both biotic (yeast extract, YE) and abiotic (MJA, metal salt) elicitors stimulated total phenol content in the shoot cultures of *S. virgata*, wherein MJA was the most effective for SAA accumulation and Ag ions for caffeic acid [23]. Moreover, MJA doubled the level of RA in hairy roots of *S. przewalskii* [25] and *S. miltiorrhiza* [18] and increased RA biosynthesis in *S. officinalis* shoots by 75% [22].

None of these reports have concerned the impact of elicitation on secondary metabolite production in *S. bulleyana* culture following elicitor treatment. Therefore, the present study, for the first time, examines the effect of harvest time and concentration of biotic (YE) and abiotic elicitors (MJA; trans-anethole, t-A; and cadmium chloride, CdCl_2_) on polyphenol production in *S. bulleyana* hairy root culture. Additionally, the hydromethanolic extract from the root culture variant with the highest phenolic acid production was subjected to further testing for its antiradical, cytotoxic and antimicrobial potential. 

## 2. Results and Discussion

### 2.1. Effect of Elicitors on the Production of Phenolic Acids in Hairy Root Culture

Although the biosynthesis of secondary metabolites in hairy root cultures is genetically controlled, production can also be influenced by environmental factors, such as biotic and abiotic elicitors. Elicitation is regarded as an important strategy for enhancing bioactive compound production in plant cultures. A number of elicitors, known to stimulate plant defense systems, are used in biotechnology and agriculture to increase secondary metabolite production [26]. Biotic elicitors are those with a biological origin. They are typically polysaccharides or are derived from pathogens, and could have a complex composition. Abiotic elicitors include hormonal (such as MJA or salicylic acid), chemical (such as metal salts: CdCl_2_, mercuric chloride, silver nitrate or cobaltous chloride or components of essential oils) or physical factors. Representatives from all these groups were selected for the present study. 

As a biotic elicitor, yeast extract was chosen. YE does not have a constant composition and is a mixture of compounds including amino acids, vitamins and minerals and various unknown compounds, which makes it difficult to determine its possible mode of action [27]. However, many studies indicate that YE has an effect on the secondary metabolism of *Lamiaceae* family members [23,28,29]. As abiotic elicitors, MJA and cadmium ions were selected, as most reports of RA elicitation concern the effect of jasmonate derivatives and heavy metal salts [23,30]. In addition, t-A, a component of essential oil of fennel and anise, was also used, as it has been reported to possibly promote secondary metabolite production in vitro: t-A treatment significantly improved ginsenoside production in *P. quinquefolium* hairy root culture [31]; however, its influence on the metabolic processes of sage species remains unknown.

Each elicitor was applied at two concentrations (100 and 200 µM Ye, 50 and 100 µM MJA, 5 and 10 µM t-A and 50 and 100 µM Cd ions) selected on the basis of their effectiveness according to the data of previous literature [30]. The optimal moment of elicitation was established; previous analyses indicate that the culture reaches a stationary phase between 35 and 40 days (data not shown). Therefore, elicitors were added at day 33, just before the plateau phase, when biomass production was generally completed, and the added compound should not have any significant negative influence on culture growth. As the reaction time of the culture varies for different exogenous compounds, four various elicitation periods (1, 3, 5 and 7 days after elicitation) were tested to determine the optimal harvest time for accumulation of phenolic acids. 

**Figure 1:**
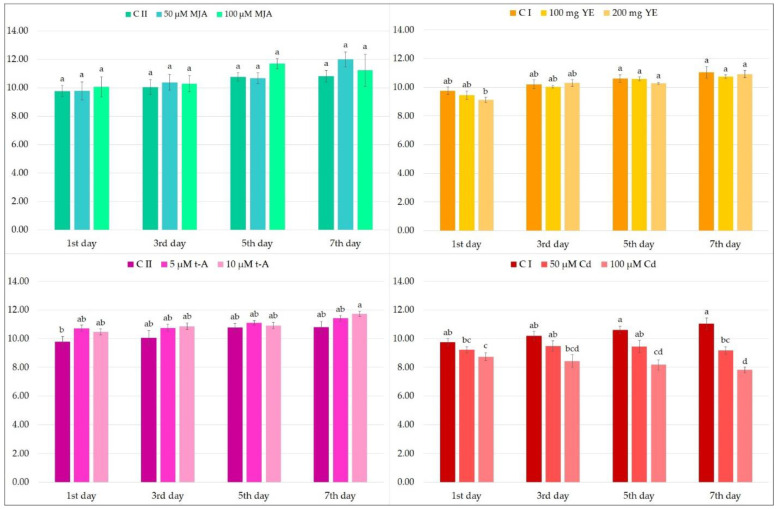
Biomass production of hairy roots (expressed as g dry weight per liter of medium) after elicitation with MJA, YE, t-A and Cd ions. CI—non treated hairy roots, CII—roots treated with 96% ethanol (solvent for MJA and t-A); the values represent the mean ± standard error of three independent experiments. Different letters indicate significant differences between samples according to the one-way ANOVA test, followed by the post hoc Tukey’s test for multiple comparison at *p* < 0.05.

Firstly, the effect of elicitor treatment on biomass accumulation was evaluated. Many studies have found elicitation to have a negative influence on the growth of, for example, *Abutilon indicum* [32], *Brugmansia candida* [33] and *Oxalis tuberosa* [34]. This is an important element in secondary metabolite biosynthesis because the productivity of the culture is related not only to content of the compound in biological material, but also to the amount of the raw material obtained at any one time. The effect of the used elicitors on dry weight accumulation is presented in Figure 1.

It can be observed that most of the compounds used have only a slight influence on the biomass accumulation (Figure 1). The exception is cadmium ions, which significantly reduced the biomass growth from the third day after elicitation when added at a concentration of 100 µM, and on the seventh day at a concentration of 50 µM.

Further analysis concerned the influence of individual biotic and abiotic elicitors on the accumulation of phenolic acids in the transformed *S. bulleyana* culture. Nine polyphenols were identified and determined in hairy root hydromethanolic extracts. The procedure for the compounds’ identification was described in an earlier publication [12].

**Figure 2:**
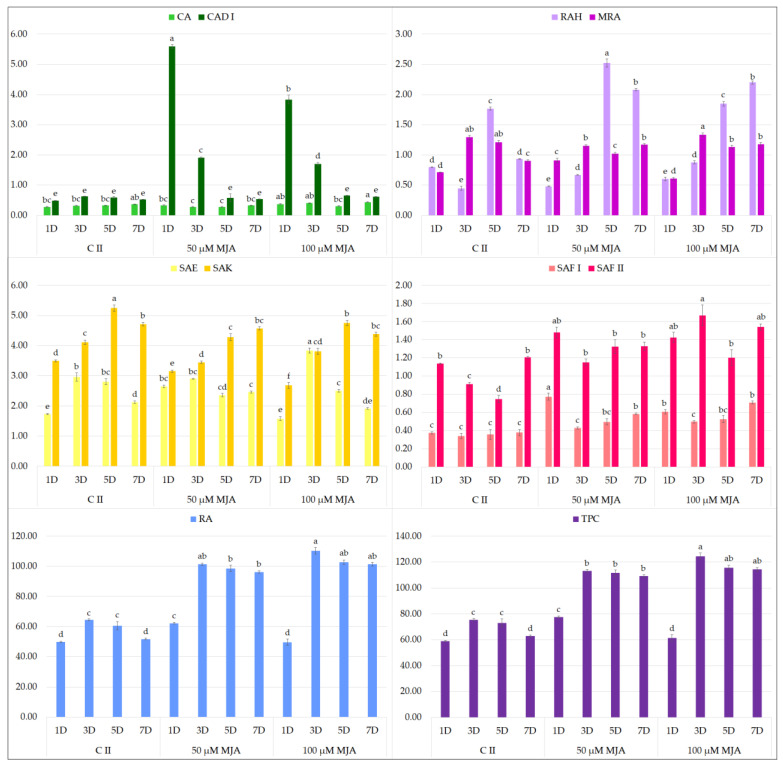
Polyphenol content 1, 3, 5 and 7 days after elicitation with 50 and 100 μM MJA (C II—untreated culture with ethanol). The values represent the mean ± standard error of three independent experiments. Different letters for the same metabolite indicate significant differences between samples according to the one-way ANOVA test, followed by the post hoc Tukey’s test for multiple comparison at *p* < 0.05. CA—caffeic acid; CAD I—caffeic acid derivative I; RAH—rosmarinic acid hexoside; MRA—methyl rosmarinate; SAE—salvianolic acid E; SAK—salvianolic acid K; SAF I and II—salvianolic acid F isomers I and II; RA—rosmarinic acid; TPC—total polyphenol content.

MJA turned out to have a particularly beneficial effect on the accumulation of polyphenols in *S. bulleyana* hairy roots (Figure 2). The addition of 100 µM MJA doubled the total phenolic acid content compared to the control, with the highest accumulation observed after 3-day exposure to elicitor (124.4 mg/g dry weight, DW). These conditions were optimal for accumulation of RA, SAE and SAF II isomer. The level of RA in the transformed roots 3 days after MJA supplementation was 110.2 mg/g DW, i.e., 13 times the level in the roots of 2-year-old plants grown in field conditions [35]. This value was also much higher than in the roots of the mother plants of most other species of sage (0.5–26 mg/g DW), a common source of this metabolite [11,13]. Similar to our results, in transformed roots of *S. przewalskii* MJA treatment doubled the RA content to 67 mg/g DW relative to the control after 3 days [25]. Three-day MJA exposure also stimulated RA accumulation in the regenerated shoot cultures of *S. virgata* by 70% to 103% depending on elicitor concentration [23]. However, such a high level of rosmarinic acid as in the elicited roots of *S. bulleyana* was obtained in only a few optimized hairy root cultures of transformed species from the *Lamiaceae*, e.g., *Ocimum basilicum* [36], *Hyssopus officinalis* [37] and *Agastache rugosa* [38].

A decrease in MJA concentration to 50 µM resulted in a reduction of total polyphenol content, but this level was approximately 50% higher than for the controls from day 3. This lower MJA concentration was found to be optimal for CAD I (caffeic acid derivative I), SAF I isomer and RAH (rosmarinic acid hexoside) production.

**Figure 3:**
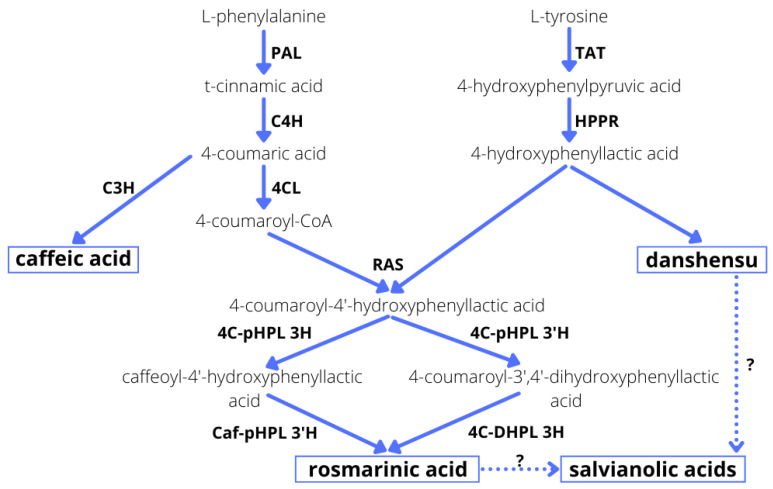
Phenolic acid biosynthesis [25,41,42]. PAL phenylalanine ammonia lyase; C4H cinnamic acid 4-hydroxylase; 4CL 4-coumaric acid CoA-ligase; TAT tyrosine aminotransferase; HPPR hydroxyphenylpyruvate reductase; C3H 4-coumarate 3-hydroxylase; RAS rosmarinic acid synthase; 3-H and 3′-H 4C-pHPL 4-coumaroyl-4′- hydroxyphenyllactate 3/3′-hydroxylases; Caf-pHPL 3′-H caffeoyl-4′-hydroxyphenyllactate 3′-hydroxylase; 4C-DHPL 3H 4-coumaroyl-3′,4′-dihydroxyphenyllactate 3-hydroxylase.

MJA is normally produced by plant cells as part of their defense response, and exogenous treatment with signal molecules can trigger a similar kind of response. Recent studies indicate that exposure to MJA regulates the biosynthesis of phenolic acids in *S. miltiorrhiza* through the induction of various transcription factors, such as MYC, MYB (myeloblastosis), WRKY, bHLH (basic helix-loop-helix) and AP2/ERF (APETALA 2/ethylene-responsive element binding factor) [39]. The genes involved in this MJA-induced plant defense metabolism have been identified as well. Briefly, RA synthesis progresses through two phenylalanine- and tyrosine-derived pathways, in which phenylalanine ammonia-lyase (PAL) and tyrosine aminotransferase (TAT) are the two first regulatory enzymes (Figure 3). These reactions produce caffeoyl CoA and 4-hydroxyphenyllactic acid, and these are condensed by rosmarinic acid synthase (RAS) to produce 4-coumaroyl-4ˊ-hydroxyphenyllactate (Figure 3). MJA is believed to activate the transcripts of PAL and C4H enzymes from the phenylpropanoid pathway, as well as TAT and HPPR from the tyrosine-derived pathway (Figure 3), thus increasing the rate of biosynthesis of RA in *S. miltiorrhiza* hairy root culture from 3.25 to 6.02% [40]. Moreover, in hairy roots of *S. przewalskii*, after treatment with MJA, various genes coding enzymes of the phenolic acid biosynthesis pathway, such as PAL, 4CL, RAS, HPPR, TAT and CYP98A14, demonstrated a significant increase in expression compared to the controls [25].

It is also worth noting that the metabolic pathways in *S. bulleyana* culture demonstrated a stepwise response to MJA elicitation. At both MJA concentrations, the level of caffeic acid increased drastically on the first day, followed by RA on the third day and then in the following days, its more complex derivatives such as RAH and MRA (methyl rosmarinate) (Figure 2). This indicates a sequence consistent with the individual stages of the phenolic acid metabolic pathway (Figure 3). This scheme may allow the duration of exposure to elicitors to be optimized with respect to individual secondary metabolites.

**Figure 4:**
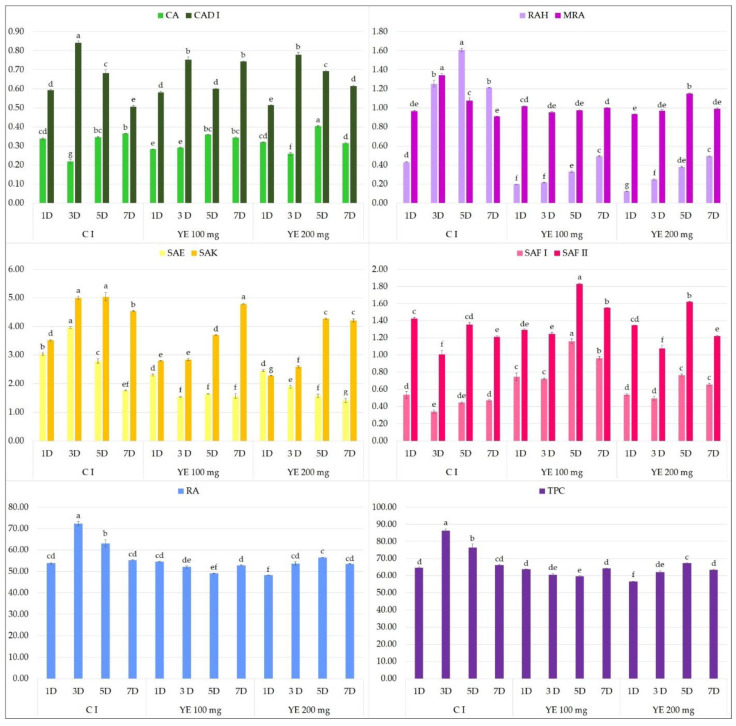
Polyphenol content 1, 3, 5 and 7 days after elicitation with 100 and 200 μM YE (C I—untreated culture). The values represent the mean ± standard error of three independent experiments. Different letters for the same metabolite indicate significant differences between samples according to the one-way ANOVA test, followed by the post hoc Tukey’s test for multiple comparison at *p* < 0.05. CA—caffeic acid; CAD I—caffeic acid derivative I; RAH—rosmarinic acid hexoside; MRA—methyl rosmarinate; SAE—salvianolic acid E; SAK—salvianolic acid K; SAF I and II—salvianolic acid F isomers I and II; RA—rosmarinic acid; TPC—total polyphenol content.

**Figure 5:**
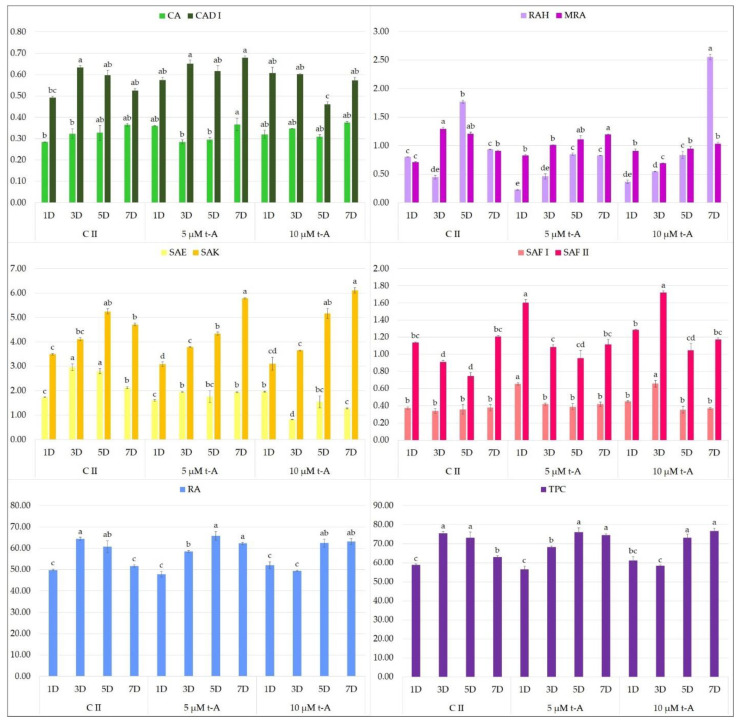
Polyphenol content 1, 3, 5 and 7 days after elicitation with 5 and 10 μM t-A (C II—non-treated culture with ethanol). The values represent the mean ± standard error of three independent experiments. Different letters for the same metabolite indicate significant differences between samples according to the one-way ANOVA test, followed by the post hoc Tukey’s test for multiple comparison at *p* < 0.05. CA—caffeic acid; CAD I—caffeic acid derivative I; RAH—rosmarinic acid hexoside; MRA—methyl rosmarinate; SAE—salvianolic acid E; SAK—salvianolic acid K; SAF I and II—salvianolic acid F isomers I and II; RA—rosmarinic acid; TPC—total polyphenol content.

In the present study, neither YE (Figure 4) or t-A (Figure 5) significantly affected the biosynthesis of total phenolic acids in transformed *S. bulleyana* roots. However, they did have some specific influence on selected individual phenolics. For example, YE increased the production of SAF I in the root culture almost threefold compared to the controls, and the production of SAF II isomer by 30% when administrated at 100 mg on day 5 (Figure 4). On the other hand, a decrease in the level of some metabolites (RA, RAH, SAE, SAK) was observed compared to the control after day 3 and 5 at both YE concentrations.

This was in contrast to previous studies of *S. miltiorrhiza* hairy root cultures, in which YE at 100 mg and 250 mg enhanced the level of RA approximately twofold, i.e., to 23 and 29 mg/g DW, respectively, from 12.5 mg/g DW [28]. Similarly, RA synthesis in hairy root culture of *Orthosiphon aristatus* increased fivefold following YE application [29]. However, YE appears to have a complex influence on RA accumulation: varying responses were observed among 11 hairy root clones of *Coleus blumei* following treatment [43]; YE supplementation reduced RA accumulation in five clones, enhanced RA accumulation in two clones and had no significant effect on four clones. Moreover, Pesaraklu et al. [39] indicate that the same elicitor could have different effects on the production of the same compounds in different species; this was confirmed by differences in the expression of key genes in the phenolic acid biosynthetic pathway between *S. officinalis* and *S. verticillata* [39]. However, any detailed tracking of these relationships is difficult since the biosynthetic pathways of individual polyphenols, other than RA, are only partially known (Figure 3).

With regard to the elicitation with t-A, the only significant effect observed for both doses was that total polyphenol content, especially RA, was maintained at an optimal level for another two days; this was observed up to day 40 of cultivation (i.e., 7 days after treatment) (Figure 5). In the natural biosynthetic cycle, i.e., in *S. bulleyana* root controls, RA content had already begun to fall by day 38.

**Figure 6:**
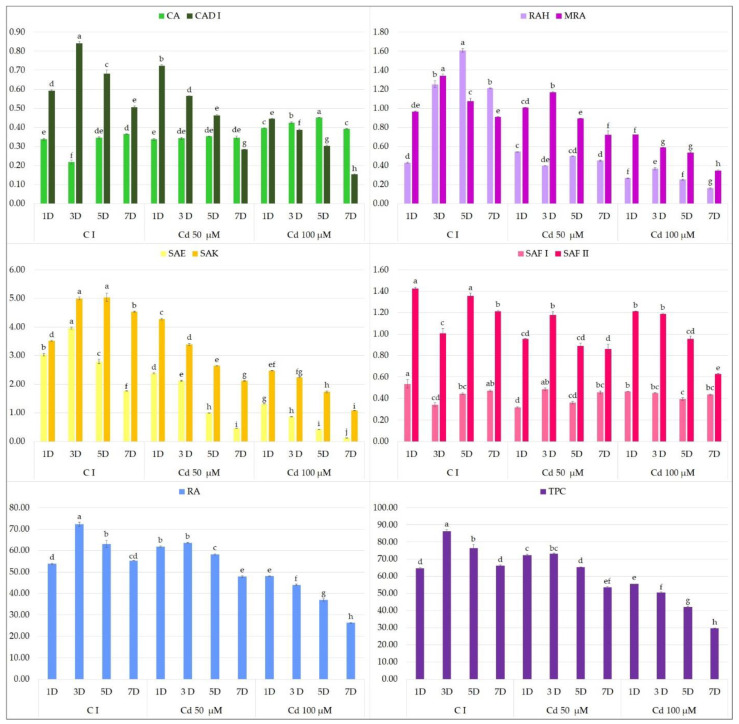
Polyphenol content 1, 3, 5 and 7 days after elicitation with 50 and 100 μM CdCl_2_ (C I—non-treated culture). The values represent the mean ± standard error of three independent experiments. Different letters for the same metabolite indicate significant differences between samples according to the one-way ANOVA test, followed by the post hoc Tukey’s test for multiple comparison at *p* < 0.05. CA—caffeic acid; CAD I—caffeic acid derivative I; RAH—rosmarinic acid hexoside; MRA—methyl rosmarinate; SAE—salvianolic acid E; SAK—salvianolic acid K; SAF I and II—salvianolic acid F isomers I and II; RA—rosmarinic acid; TPC—total polyphenol content.

Cadmium treatment had a negative effect on polyphenolic compound production in *S. bulleyana* hairy root culture at both concentrations (Figure 6). The reaction was visible as early as 1 day after elicitation at a concentration of 100 µM, and from day 3 in the case of 50 µM. Ultimately, exposure to cadmium at a concentration of 100 µM lowered the total level of polyphenols in the culture from 14% on day 1 to 55% on day 7, while the total phenolic acid content was only 29.6 mg/g DW. In the case of the lower concentration, this effect was only observed after 3 days of exposure, with values ranging from 14.6 to 19.2%. One compound that did not appear to react significantly on the presence of CdCl_2_ was the SAF I isomer.

The adverse effect of cadmium on productivity may stem from its detrimental effect on culture growth, which translated into lower bioactive compound production. Both biomass accumulation and polyphenol production were inversely proportional to the concentration of cadmium ions. Although heavy metals can be used as stress factors to intensely promote secondary metabolite production in plants, they can also induce a hypersensitive response, leading to cell death [44]. This mechanism may arise through the displacement of iron and other divalent cations from proteins: the free ions can induce oxidative injury via Fenton reactions [45]. Zhao et al. [46] report that heavy metals such as cadmium successfully increase tanshinone production in *S. miltiorrhiza* cell culture. In *Phoenix dactylifera* culture, treatment with CdCl_2_ at the concentration significantly higher than in the present experiment (50 mg/L) resulted in double the total flavonoid content compared to controls, with no negative effect on the fresh or dry mass. Our present findings indicate that *S. bulleyana* culture has significant sensitivity to heavy metals, especially following prolonged exposure. Our results corroborate those of Chodisetti et al. [44] on *Gymnema sylvestre* culture, where treatment with 2 µM CdCl_2_ resulted in an initial short-term stimulation of secondary metabolite production followed by a rapid decrease in culture biomass and gymnemic acid accumulation (almost by half) after exposure longer than 24 h.

### 2.2. Biological Potential of the Extract Obtained from MJA-Elicited Hairy Root Culture

Finally, the biological potential of the optimized, highly productive *S. bulleyana* culture was estimated. The study focused on the areas of activity particularly related to RA, i.e., the predominant metabolite of hairy root extract.

**Figure 7:**
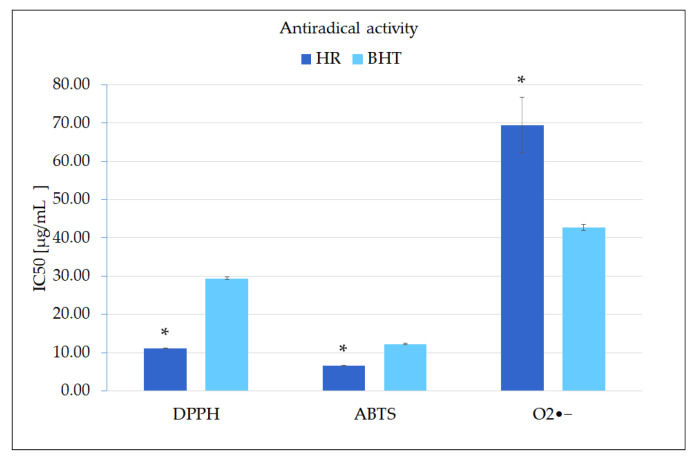
Antiradical activity *S. bulleyana* extract expressed as IC_50_ values (µg/mL). The values represent the mean ± standard error of three independent experiments. * statistically significant differences vs. control (BHT) for the same test according to the Kruskal–Wallis test (*p* < 0.05).

The most frequently used methods for assessing the antioxidant activity of plant extracts include: the reduction of transition metal ions tests, free radical scavenging assays and inhibition of lipid peroxidation tests [47]. To determine the antioxidant effect of *S. bulleyana* hairy root extract, three antiradical assays were used, including synthetic and natural, both nitrogen and oxygen-centered radicals (Figure 7). These methods are based on the SET (single electron transfer) or HAT (hydrogen atom transfer) mechanism, where an electron or a hydrogen atom is transferred from the antioxidant to radical [47]. The reduction of the purple-colored solution of DPPH radical in the presence of hydrogen-donating antioxidants is characterized by a color change to yellow, while changes in the concentration of the ABTA radical caused by the reaction with the antioxidant result in the discoloration of its green-blue solution. In the superoxide anion scavenging assay, the radical content is assayed through reduction of NBT. The decrease in the extent of NBT reduction, measured by the absorbance of the reaction mixture, correlates with the ability of the antioxidant to scavenge of superoxide radical [47].

Since the extract of the transformed roots treated with MJA contains a high level of phenolic compounds, it is not surprising that it exerted strong scavenging potential towards different free radicals (Figure 7). In the DPPH assay, the extract exhibited very strong scavenging activity with IC_50_ = 11.1 µg/mL, i.e., three times higher than the synthetic industrial antioxidant BHT. Moreover, the antiradical potential determined by ABTS assay (IC_50_ = 6.5 µg/mL) was also more effective than BHT. 

Antiradical activity in numerous species of sage has often been measured towards synthetic radicals. The appropriate analytical methods are inexpensive and allow quick estimation of the antioxidant potential of raw material. The DPPH scavenging assay found the root and shoot extracts of several sage species to have IC_50_ values ranging from 2.0 to over 500 µg/mL [48,49,50]. Only a few species, such as *S. schlechteri* (2 µg/mL) [48] and *S. elegans* (10.7 µg/mL) [50], also showed greater activity to that found in the present study.

In the case of superoxide anion (O_2_^•−^), the investigated *S. bulleyana* hairy root extract had a slightly weaker activity (IC_50_ = 69.5 µg/mL) than that of the control (BHT); however, its potential against this radical was still similar or greater compared to other species of sage [51,52]. Quenching capacity towards O_2_^•−^ is especially important because it plays an essential role in the oxidative damage in the human body and hence has significance in generating oxidative stress-related civilization diseases.

Importantly, our results obtained for the elicited hairy roots of *S. bulleyana* were significantly higher than those determined previously under the same conditions for the roots of two-year-old field-grown plants of this species [35]. The traditionally grown plants demonstrated IC_50_ values that were twice as high for O_2_^•−^ scavenging, three times higher for DPPH and more than five times higher for ABTS compared to the present findings; this is undoubtedly related to the significant increase in the level of phenolic acids observed in the elicited culture compared to the roots of traditionally grown plants (5.5-fold increase). A key role in antioxidant effect of the extract may be played by RA, whose activity against the DPPH radical was estimated with IC_50_ of 12.4 µg/mL [53]. Moreover, while no study currently exists to confirm this hypothesis, other polyphenols identified in the extract may also be involved in its antioxidant activity.

Increasingly, plant extracts with strong antioxidant properties are considered to support the fight against cancer. The cytotoxicity of extracts from the underground and aerial parts of different sage species have been reported in a number of studies. Methanolic or hydromethanolic extracts of *S. eremophila* were found to be effective against breast cancer MCF-7 cell lines [49]. Ethanolic and acetone extracts from *S. miltiorrhiza* roots, as well as of *S. officinalis* roots and leaves, have demonstrated a high cytotoxic capacity against HepG2 cell lines [54]. In addition, methanolic extracts from *S. ceratophylla* roots at 0.1 mg/mL were found to be twice as toxic towards HepG2 cells than the extract from the aerial parts, reducing cell viability to 30.9% and 75.3% vs. the control, respectively [55]. Therefore, the present study aimed at initial evaluation of the cytotoxic potential of the *S. bulleyana* extract against three human cancer cell lines: AGS, LoVo and HeLa. 

**Figure 8:**
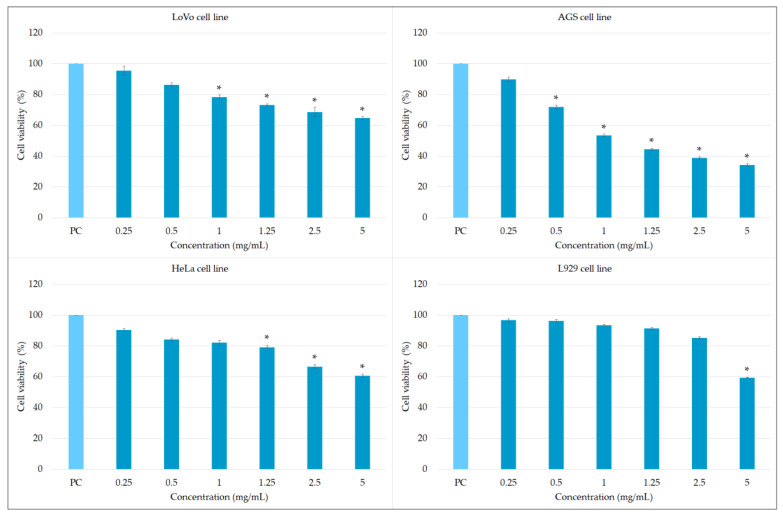
Cytotoxic effect of *S. bulleyana* extract towards the LoVo, AGS, HeLa and L929 cell lines. The cell viability was calculated for four experiments, including three replicates for each compound. Complete RMPI-1640 medium (cRPMI) was used as a positive control of cell viability (100% viable cells) and 0.03% H_2_O_2_ as a negative control of cell viability (5% for LoVo, 2.2% for AGS, 3.2% for HeLa, 3.3% for L929). *—determination of statistical significance for untreated cells vs. cells treated with tested plant extract for the same cell line according to the Kruskal–Wallis test (*p* < 0.05).

According to the results, the hairy root extract demonstrated statistically significant cytotoxic activity against LoVo cells in the concentration range 1–5 mg/mL and against HeLa cells at 1.25–5 mg/mL: the treatments resulted in 22–46.5% and 21–39% of dead cells, respectively (Figure 8). The cytotoxic potential against the AGS cell line was even higher (in the concentration range 0.5–5 mg/mL, 29–66% of dead cells) (Figure 8).

Of particular relevance are the cytotoxic effects of *S. bulleyana* roots against LoVo and AGS cell lines since both lines are derived from gastrointestinal cancer cells: gastric adenocarcinoma epithelial cells and human colon epithelial cells, respectively. The tested extract can reach these cells after oral administration in unchanged form, before undergoing degradation in the liver. The first-pass effect is a key reason for the differences observed between results obtained in vitro on cell lines and in vivo on living organisms; in such cases, formulations given *per os* may undergo multiple metabolic changes, and can often be inactivated or transformed to less active forms. Moreover, when taken orally, it is possible to achieve much higher concentrations in the gastrointestinal tract than anywhere else in the body.

The cytotoxic effect of *S. bulleyana* extract may be related to the presence of RA in the hairy roots. It has been shown that RA can inhibit proliferation and induce apoptosis of liver, prostate, breast, ovarian, lung, leukemia and skin melanoma cancer cell lines [4]. It has also been demonstrated that RA increases the sensitivity of tumor cells to chemotherapeutic drugs [4]. Recent studies have found that treatment with RA resulted in an increased apoptosis rate in cell cultures of gastric cancer cells and human colon carcinoma-derived cell lines [5,56,57]. A rich-in-RA rosemary extract was able to induce G2/M cell cycle arrest in gastrointestinal cancer cell lines with IC_50_ values 4.1, 1.8 and 1.3 mg/mL after 24, 48 and 72 h of exposure, respectively [57]. These results were similar to those obtained for the studied *S. bulleyana* root extract. 

Currently, due to their low content in biological materials, no reports exist on the cytotoxic or cytostatic activity of the salvianolic acids which have been identified in *S. bulleyana* extract, such as salvianolic acid E, K or F. On the other hand, numerous studies document the strong anticancer activity of more common phenolic acids with similar structure, e.g., salvianolic acid A (SAA) and B (SAB) [58]. Recently, SAB was found to inhibit the growth and angiogenesis of oral cancer cells, whereas SAA prevented their metastasis by inhibiting the pathway controlling the expression of matrix metallopoteinase-2. Furthermore, SAB appeared to inhibit the growth of head and neck squamous cell carcinoma cells, induce apoptosis and reduce the formation of solid tumors by reducing the expression of *cyclooxygenase-2,* while SAA inhibited the growth of mouse lung cancer cells [58]. In addition, SAB demonstrated a synergistic effect with chemotherapeutic agents such as 5-fluorouracil in the inhibition of LoVo cell growth in vivo in a dose-dependent manner [59]. Similar properties would also be expected for other salvianolic acid derivatives; these should be isolated in future studies to confirm their pharmacological potential.

Before biocompounds and plant extracts can be used in treatment or prophylaxis in humans, the FDA (Food and Drug Administration) require them to be subjected to biocompatibility studies involving L929 mouse fibroblasts (according to ISO 10993-5). Therefore, our in vitro studies examined the potential cytotoxic effect of the tested plant extract towards L929 cells to determine its safe concentration based on the MTT reduction assay. The hydromethanolic extract of *S. bulleyana* diminished the growth of L929 fibroblasts only at the highest tested concentration (5 mg/mL) (Figure 8); lower concentrations did not reduce cell viability statistically significantly compared to the control. These findings indicate that the analyzed extract is safe for normal cells. The negative control (0.03% H_2_O_2_) under the same conditions reduced cell viability to 2% vs. the control. 

Infectious diseases still represent a significant cause of morbidity and mortality in humans. Even though a number of new antimicrobial drugs have been introduced in the last years, resistance to these drugs by microorganisms still remains a problem [60]. A number of plant extracts show antimicrobial and antifungal activity, and hence they might be candidates for therapeutic formulations [61,62,63]. Therefore, the present study made a preliminary assessment of the activity of *S. bulleyana* extract against the Gram-positive bacteria *Staphylococcus aureus* and *S. epidemidis*, the Gram-negative bacteria *Escherichia coli* and *Pseudomonas aeruginosa* and the fungal strains *Candida albicans* and *C. glabrata*.

*S. bulleyana* extract presented moderate antibacterial and antifungal activity: the minimal inhibitory concentration (MIC) and minimal bactericidal concentration (MBC)/minimal fungicidal concentration (MFC) are shown in Table 1 along with MIC/MBC/MFC for standard antibiotics (gentamicin and amphotericin B). The strongest bactericidal activity was found against the Gram-positive *S. epidemidis*, with MIC equal to 1.25 mg/mL and MBC as high as 10 mg/mL (Table 1). The tested extract showed a slightly weaker bacteriostatic effect against Gram-negative bacteria, with MIC of 2.5 mg/mL for *P. aeruginosa* and *E. coli* with a slightly greater bactericidal effect (MBC) equal to 2.5–5 mg/mL. These results are consistent with other reports on *Salvia* species. *S. africana* and *S. mexicana* extracts were found to exhibit similar antibacterial activity as our *S. bulleyana* extract against *S. epidermidis*, as well as slightly stronger effects towards *S. aureus* (MIC/MBC: 0.6–1.2 mg/mL/1.25 mg/mL) and weaker towards Gram-negative bacteria (7.5->10 mg/mL/7.5->10 mg/mL) [64]. The MICs of methanol extracts from *S. xanthocheila*, *S. santolinifolia*, *S. limbata* shoots against six different Gram-negative and Gram-positive bacteria were also in the range of 1.25–5 mg/mL; however, in the same experiment, *S. sclarea* and *S. eremophila* showed a much stronger effect against *S. epidemidis* (MIC 0.3 mg/mL) [49]. The hydromethanolic extracts of *S. cadmica* shoot (1.25–5 mg/mL) also demonstrated similar activity against selected Gram-positive bacteria [52], while the root extract was more active against *Staphylococcus* strains (0.156–2.5 mg/mL). 

Additionally, the tested extract showed relatively weak antifungal effect against *Candida* species, with MIC/MCF 10 mg/mL (Table 1). Similar or even weaker anti-*Candida* activities were reported for *S. tigriana* ethanolic extracts (MIC 6.25–25 mg/mL) [65] or *S. verticillata* (10 mg/mL) [66]. The study by Martin et al. [67] revealed that extracts of *S. officinalis* showed a weak potential against the majority of *Candida* species. However, hydromethanolic extract from *S. cadmica* (0.312–1.25 mg/mL) [52], *S. sclarea* (0.2–1 mg/mL) [68] or *S. viridis* (1.25–1.5 mg/mL) [69] exhibited significantly stronger activity.

In conclusion, the antimicrobial activity of *S. bulleyana* extract was found to be too weak to be used alone, but it may be of importance in multicomponent therapy in order to achieve a synergistic effect with synthetic therapeutic agents.

## 3. Materials and Methods

### 3.1. Hairy Root Culture

Aseptically germinated shoots of *S. bulleyana* were infected with a *Rhizobium rhizogenes* bacterium (A4). They were then maintained on hormone-free WP (Woody Plant) [70] medium with 30 g/L sucrose at 25 °C in the dark [12]. The clone used in the study (C4) was selected on the basis of its growth rate and production of polyphenolic compounds [12]. 

All of the experiments in this study were carried out in 300-mL Erlenmeyer flasks on a shaker set in the dark at 70 rpm. Each flask, containing 80 mL liquid ½SH (Schenk and Hildebrandt) [71] medium with a half measure of vitamins and 30 g/L of sucrose, were inoculated with 0.426 g fresh weight (FW) and 0.057 g dry weight (DW) of roots from a 5-week-old shake-flask culture. The optimal basal medium for the cultivation of this culture was selected on the basis of separate experiments (data not shown). All of the growth media used in the study were purchased from Duchefa Biochemie (Haarlem, The Netherlands).

### 3.2. Elicitation Experiment

All of the elicitors used in the experiment were added on day 33 of culture. To study the effect of YE and Cd (Sigma-Aldrich, Darmstadt, Germany), the concentrated, sterilized solution was added by pipette with a sterilized tip to the hairy root culture of *S. bulleyana* to final concentrations of 100 and 200 mg/mL (YE) and 50 and 100 µM (Cd). Concentrated MJA and t-A (both from Sigma–Aldrich, Darmstadt, Germany) solutions were prepared by dissolving reagents in 96% ethanol. Elicitor from stock solution was added (10 µL per flask) using 0.22 µM filter to reach the two final concentrations: 50 and 100 µM (MJA) and 5 and 10 µM (t-A). The hairy roots were harvested 1, 3, 5 and 7 days after elicitation, and their biomass and secondary metabolite accumulation was evaluated. Plant material from the control was harvested on the same days as the treated roots. The controls were non-treated hairy roots for YE and CD treatment (control I, CI) and 96% ethanol (the solvent for the elicitors; 10 µL per flask) for MJA and t-A treatment (control II, CII).

All of the cultures were maintained at temp. 26 ± 2 °C on a rotary shaker at 70 rpm under darkness. For plant material from all of the treatments and corresponding control cultures, the dry weight was determined (expressed as g/L of culture), as well as the total polyphenolic compound content. All of the experiments were replicated three times.

### 3.3. Evaluation of Hairy Root Growth and Quantification of Phenolic Acids

The hairy roots were harvested after 1, 3, 5 and 7 days of treatment (on days 34 and 36, 38 and 40 of culture) from the shake flasks and washed three times with distilled water, and then frozen and lyophilized until constant dry weight.

Lyophilized powdered material was mixed with solution methanol: water (4:1, *v*/*v*) and sonicated for 15 min. The extraction was performed according to Wojciechowska et al. [12]. The extraction was repeated twice. The combined extracts were evaporated and stored as dry extracts at 4 °C until analysis. Phenolic acids were determined by HPLC. Separation was performed on an Ascentis Express C-18 column (7.5 cm × 4.6 mm, 2.7 μm; Supelco, Bellefonte, PA, USA), as described previously [12]. Compounds were detected at λ = 325 nm and their content was expressed in mg/g dry weight (DW). The total phenol content was calculated as the sum of the contents of all of the quantified phenolic acids.

### 3.4. Biological Activity Study

The hydromethanolic extract obtained from the root culture subjected to 3-day elicitation with 100 µM MJA was subjected to antiradical, antimicrobial and cytotoxic assays. Just before the analyses, the dry extract stored in a refrigerator (4 °C) was dissolved for testing. Dissolution was performed in a water–methanol (1:4) solution for phytochemical and antioxidant testing in a cell culture medium for cytotoxic testing and in 0.5% DMSO diluted with microbial culture medium for microbiological testing. 

#### 3.4.1. Antiradical Activity

The ability to scavenge reactive radicals were evaluated using DPPH assay according to Grzegorczyk–Karolak and Kiss [51]. After 30 min of incubation, the absorbance of the solution was measured at 517 nm (Beijing Reyleigh Corp., Beijing, China). The activity towards ABTS was evaluated as previously described [72]. The absorption at 734 nm was recorded after 10 min. The radical scavenging properties towards O_2_^•−^ were evaluated using nitroblue tetrazolinum assay [51]. The absorption at 560 nm was measured after 5-minute incubation. The antiradical potential was expressed as IC_50_ (µg/mL of dry extract), i.e., the concentration needed to reduce of initial concentration of radicals by 50%. Butylated hydroxytoluene (BHT) purchased from Sigma–Aldrich (Darmstadt, Germany) was used as a control antioxidant. 

#### 3.4.2. Cytotoxic Activity

In vitro cytotoxicity testing was performed using human HeLa (CCL-2, American Type Culture Collection (ATCC), Rockville, MD, USA) cervix adenocarcinoma epithelial cells, human AGS (CRL-1739, ATCC, Rockville, MD, USA) gastric adenocarcinoma epithelial cells, human colon epithelial LoVo (CCL-229™, ATCC, Rockville, MD, USA) and normal mouse fibroblasts L929 (LGC Standards, Middlesex, UK). The L929 cell line is required for testing by ISO norm 109935. 

Cell viability, assessed by trypan blue assay, ranged from 93–95%. All cell lines were cultivated in 25 cm^2^ tissue culture flasks in RPMI-1640 medium supplemented with 10% heat-inactivated Fetal Bovine Serum (FBS), penicillin (100 U/mL) and streptomycin (100 μg/mL) (all from Biowest, Minneapolis, MN, USA), under standard conditions (37 °C, 5% CO_2_). The cell culture medium was replaced two or three times per week to keep cells in log phase, and the confluent cell monolayer was treated with 0.25% trypsin solution to passage. 

The metabolic activity of the L929, AGS, HeLa and LoVo cells was tested after application of the *S. bulleyana* hydromethanolic extracts at concentrations of 0.25, 0.5, 1.25, 2.5 and 5 mg/mL, as described by Weremczuk-Jeżyna et al. [73]. Cell metabolism was estimated on the basis of MTT reduction, as described by Kamizela et al. [74]. The MTT reduction assay is recommended by the Food and Drug Administration (FDA) and the International Organization for Standardization (IOS).

#### 3.4.3. Antimicrobial Potential

The antibacterial and antifungal activities of *S. bulleyana* extract were determined by the broth microdilution assay according to The European Committee on Antimicrobial Susceptibility (EUCAST) recommendations. The antimicrobial activity, expressed as mg/mL, was evaluated based on the minimal inhibitory concentration (MIC) and minimal bactericidal concentration (MBC) or minimal fungicidal concentration (MFC), towards the following reference strains from the American Type Culture Collection (ATCC): Gram-positive *Staphylococcus aureus* ATCC 29,213 and *Staphylococcus epidermidis* ATCC 12,228; Gram-negative *Escherichia coli* ATCC 29,212 and *Pseudomonas aeruginosa* ATCC 27,853; the fungi *Candida albicans* ATCC 10,231 and *Candida glabrata* ATCC 2001. Mueller–Hinton (MH) liquid medium (pH~7.2) was used for the bacteria while liquid medium without phenol red RPMI-1640 (pH~7.2) was used for the fungal strain. Briefly, the inocula, prepared according to the McFarland scale, containing 1 × 10^8^ colony forming units (CFU/mL), were added at a volume of 10 μL to the extract solution and incubated for 24 h at 37 °C (bacteria) or 48 h at 37 °C (fungi). Amphotericin B was used as the antifungal standard and gentamicin as the antibacterial standard.

### 3.5. Statistical Analysis

All of the experiments were performed in triplicate and the results are given as mean values ± SE. The influence of various treatments on biomass accumulation and polyphenolic compound content was analyzed by one-way analysis of variance (ANOVA) followed by Tukey’s post hoc test, and the biological activity was compared between sample and control by the Kruskal–Wallis test. The results were regarded as statistically significant at a probability level of *p* < 0.05. All of the calculations were performed using STATISTICA 10 PL software (Statsoft, Kraków, Poland). 

## 4. Conclusion

This work is the first report on the impact of elicitation on the production of polyphenols in the hairy root culture of *S. bulleyana*. Among the four different elicitors tested, MJA was found to be the most effective one, doubling the biosynthesis of phenols after 3 days of treatment at 100 µM. Its effectiveness might be connected with its regulatory impact on the activity of enzymes in biosynthesis pathways. The obtained elicited culture is, in terms of productivity, among the best biotechnological sources of RA and it provides an effective tool for the industrial-level production of RA and other phenolic acids from *S. bulleyana.* On the other hand, the *S. bulleyana* hairy roots turned out to be extremely sensitive to the negative effects of cadmium ions, which suggests that high metal salts concentrations should be avoided if high culture productivity is to be maintained. Moreover, the extract from *S. bulleyana* roots elicited by MJA demonstrated very strong antiradical activity and significant cytotoxic effects against the gastric cancer AGS cell line, while being relatively safe for normal mice fibroblasts. Such effects merit further analysis for potential application towards gastrointestinal tract malignancies.

## Figures and Tables

**Table 1:** Antimicrobial activity of *S. bulleyana* extract shown as minimal inhibitory concentration (MIC) and minimal bactericidal concentration (MBC) or minimal fungicidal concentration (MFC).

Microorganism	*S. bulleyana* Extract		Gentamicin	Amphotericin B
	MIC (mg/mL)	MBC/MFC (mg/mL)	MIC = MBC/MFC (µg/mL)	
Gram-negative bacteria				
*Pseudomonas aeruginosa* ATCC 27853	2.5	5	<8	-
*Escherichia coli* ATCC 25922	2.5	2.5	<4	-
Gram-positive bacteria				
*Staphylococcus aureus* ATCC 29213	2.5	10	<2	-
*Staphylococcus epidermidis* ATCC 12,228	1.25	10	<2	-
Fungi				
*Candida albicans* ATTC 10231	10	10	-	<1
*Candida glabrata* ATCC 2001	10	10	-	<1

Gentamicin and amphotericin B, board-spectrum antibiotics, used as antibacterial and antifungal reference substances, respectively; (-) not tested. ATCC—American Type Culture Collection.

## Data Availability

Data is contained within the article.

## Sample Availability

Samples of the compounds are not available from the authors.
